# *Scutellaria lateriflora* Extract Supplementation Provides Resilience to Age-Related Phenotypes in *Drosophila melanogaster*

**DOI:** 10.3390/ijms27010461

**Published:** 2026-01-01

**Authors:** Dani M. Long, Jesus Martinez, Amala Soumyanath, Doris Kretzschmar

**Affiliations:** 1 BENFRA Botanical Dietary Supplements Research Center, Oregon Health & Science University, Portland, OR 97239, USA; 2 Oregon Institute of Occupational Health Sciences, Oregon Health & Science University, Portland, OR 97239, USA; 3 Department of Neurology, Oregon Health & Science University, Portland, OR 97239, USA

**Keywords:** herbal supplements, aging, sleep fragmentation, neurodegeneration

## Abstract

The human lifespan has increased dramatically over the last few decades; however, reaching older age increases the risk of age-related diseases and ailments. To extend the healthspan, many have turned to supplements, including plant-based remedies used in traditional medicine, to promote healthy aging. One of these is *Scutellaria lateriflora* L. (*S. lateriflora*), native to North America, which has traditionally been used to treat anxiety, stress, and insomnia. However, clinical trials addressing its effects are very limited. Furthermore, plant material is intrinsically complex, and the preparation method affects the composition of extracts. We therefore used *Drosophila* to test whether *S. lateriflora* can confer resilience against age-related sleep and mobility deficits, using aqueous (SLAq) and ethanol extracts (SLE). Whereas both SLE and SLAq improved mobility, only SLE reduced sleep fragmentation in older males. By testing several flavonoids present in *S. lateriflora*, we found that the beneficial effects on mobility were mainly due to baicalin, whereas sleep was improved by a wogonin mix. Since neither the extracts nor the compounds extend the lifespan, this suggests that they improve neuronal health and function and do not generally slow down the aging process. This was supported by our finding that neuronal degeneration was reduced by *S. lateriflora* (SL) supplementation.

## 1. Introduction

Aging is associated with declines in locomotor and cognitive function [1,2], as well as increases in sleep fragmentation [3,4,5] and neurodegeneration [6]. Sleep disruptions alone can lead to learning and memory impairments [7], increase anxiety [8], and even increase the risk of neurodegeneration [9,10]. While some medications exist to help with some of these symptoms, none address all, and they are often accompanied by unwanted side effects or the possibility of drug interactions. Sleep medications, especially with long-term use, come with the risk of dependency and an increased risk of dementia [11,12]. At the same time, medicines for cognitive decline, such as cholinesterase inhibitors, have mixed results regarding efficacy but have many reported side effects [13,14]. This has led to an increased interest in nutritional supplements to help build resilience against age-related cognitive and functional decline. Many supplements sold alongside vitamins and minerals are composed of botanicals that have been used in traditional medicine [15,16].

One such botanical is *Scutellaria lateriflora* L. (also known as American Skullcap), a member of the mint family (Lamiaceae) native to North America [17,18]. Traditionally used as a nerve tonic, sedative, and anticonvulsant, among others [16], *S. lateriflora* has been used as a mild sedative/relaxant for over 200 years [18,19,20]. Currently, it is used as a botanical dietary supplement for anxiety, and better sleep patterns and quality of sleep have also been reported [21]. To date, only four clinical human trials have been published, with two reporting decreased anxiety and improved mood [22,23] and another describing improved sleep parameters in response to *S. lateriflora* treatment [24]. The fourth tested the effects of *S. lateriflora* in gingivitis [25]. Although these studies differed in participant selection, product preparation, dosage, treatment delivery, duration, and measured outcomes, all studies reported that *S. lateriflora* was well tolerated among participants. Several studies have reported on the constituents of many *Scutellaria* species and extracts. Flavonoids are the main components of several *Scutellaria* species, including *S. lateriflora* and *S. baicalensis*. However, the relative quantities differ between *Scutellaria* species and the parts of the plant in which they are contained [26,27,28]. The flavonoid components of *S. lateriflora* aerial parts have been shown to possess anti-bacterial, anti-viral, antioxidant, anti-inflammatory, anti-invasive, neuroprotective, hepatoprotective, and pro-apoptotic properties [16].

In the present study, we aimed to evaluate the effects of *S. lateriflora* and its major flavonoids on sleep and cognition-related outcomes. Like humans, *Drosophila melanogaster* show age-related changes in locomotion, cognition, and sleep fragmentation [29]. We therefore used *Drosophila* to investigate the effects of two different *S. lateriflora* preparations on age-related phenotypes. To identify which flavonoids may provide resilience to age-related functional decline, we quantified several flavonoids in both of our extracts and tested those in our model at doses corresponding to their concentrations in the most active extract.

## 2. Results

Many people start taking supplements with age to improve their health in the later stages of life [30]. To examine the effects of *S. lateriflora* (SL) extracts on aging in the *Drosophila* model system, male and female flies were fed a standard *Drosophila* diet until they were four weeks old (middle age). They were then switched to an extract-supplemented diet or a corresponding control diet. Hydroethanolic extract (SLE) or an aqueous extract (SLAq) of *S. lateriflora* was added to the standard fly diet at multiple concentrations. For SLE, the concentrations used were 1, 2.5, and 5 mg/mL, while, for SLAq, we used 1 and 2.5 mg/mL. We chose not to test SLAq at 5 mg/mL, as flies did not tolerate 5 mg/mL SLE well, and it shortened the median lifespan (see below). The control diet contained the same amount of vehicle (water) as was used for each concentration of the extracts. Flies were maintained on a supplemented or control diet for two weeks before behavioral (cognitive/locomotor and sleep) assessments.

### 2.1. S. lateriflora (SL) Treatment Improves Phototaxis

**Figure 1:**
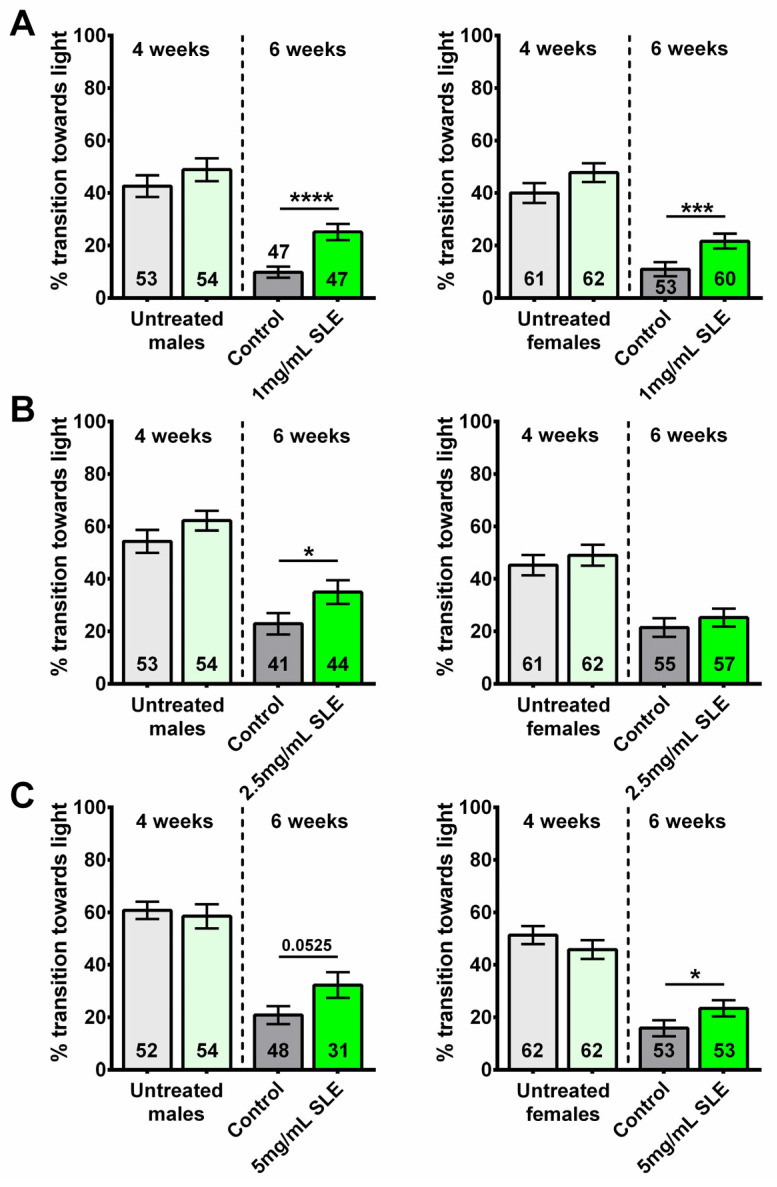
SLE treatment can provide resilience to age-related cognitive/locomotor decline. Performance declines with age in all groups when comparing the flies before treatment (4 weeks old, light green and light gray, left of the dotted line) with untreated 6-week-old flies (controls, gray). However, after a 2-week treatment with (**A**) 1 mg/mL SLE (green), males (**left**) and females (**right**) perform better than the controls. (**B**) Treatment with 2.5 mg/mL SLE (green) also improved phototaxis but only in males (**left**). (**C**) Treatment with 5 mg/mL SLE (green) improved cognitive/locomotor performance in females and males but only reached statistical significance in females (**right**). Data are represented as the mean ± SEM of three independent experiments per age and treatment group. The total number of flies analyzed is denoted in each column. Due to the values not being normally distributed, as determined by the D’Agostino–Pearson test, the non-parametric Mann–Whitney test was used to determine statistical significance between groups. * *p* < 0.05, *** *p* < 0.001, **** *p* < 0.0001.

Fast phototaxis is an assay used to evaluate cognitive and locomotor performance in *Drosophila*. During this assay, flies must detect the light source, orient themselves, and run toward the light in quick succession; performance in this assay declines with age [31]. At 4 weeks of age, prior to treatment, we assigned half of the flies to the control or treatment groups. Although selected randomly, we confirmed that both groups indeed performed equally well in the phototaxis assay (Figure 1A–C, left panel). After 2 weeks of supplement/vehicle treatment, the cognitive/locomotor performance of the now 6-week-old flies was assessed again. As previously shown [32,33], the performance of 6-week-old flies was lower than that of 4-week-old flies across all groups. However, differences were found between the treatment and control groups at 6 weeks of age. Two-way ANOVA analyses, including comparisons of age for all the phototaxis data, are shown in Supplementary Table S1.

Flies treated with 1 mg/mL SLE showed an improved performance compared to age- and sex- matched controls (Figure 1A). Compared to their respective controls, 6-week-old males treated with 2.5 mg/mL SLE also performed significantly better; however, no significant differences were observed between SLE-treated and control females (Figure 1B). The opposite was seen with 5 mg/mL, where SLE-treated females showed an improved performance compared to controls, but not the males (Figure 1C). Comparing the different concentrations, we found that 2.5 mg/mL and 5 mg/mL are efficient in males and 2.5 mg/mL is efficient in females (Supplementary Figure S1A,B).

**Figure 2:**
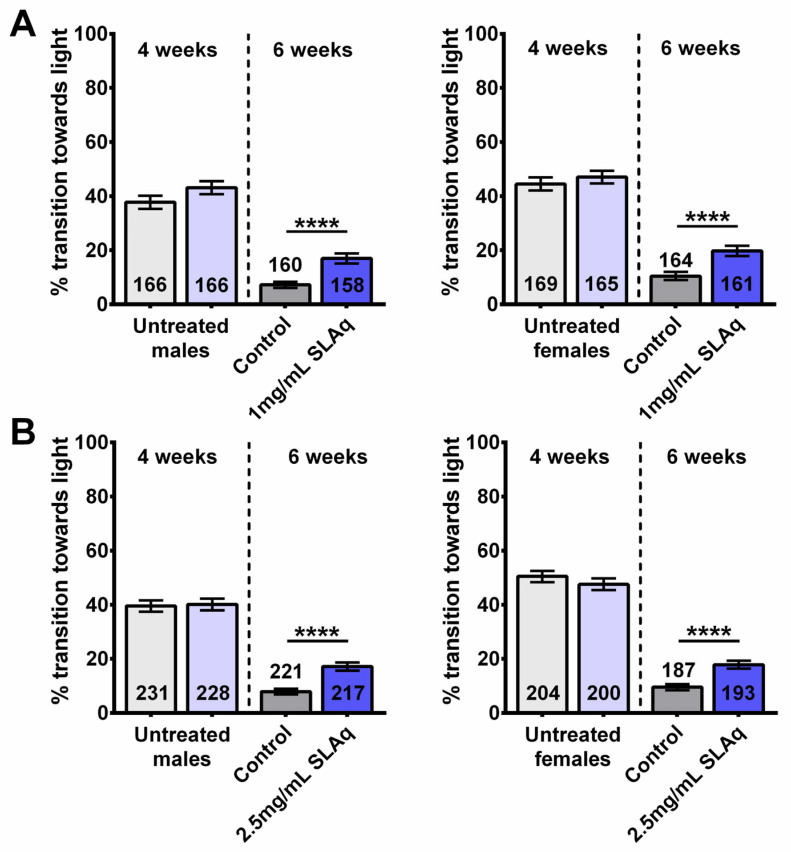
SLAq treatment provides resilience to age-related cognitive/locomotor decline. Prior to treatment, 4-week-old flies performed similarly (light gray and light blue, left of the dotted line), and performance declined with age compared to 6-week-old controls (gray). After the 2-week treatment, 6-week-old flies with (**A**) 1 mg/mL SLAq or (**B**) 2.5 mg/mL SLAq (blue) showed improved performance when compared to controls. Males are shown on the left, females on the right. Data are represented as the mean ± SEM of 3 independent experiments per age and treatment group. The total number of flies analyzed is denoted in each column. Due to the values not being normally distributed (D’Agostino–Pearson test), the non-parametric Mann–Whitney test was used to determine statistical significance between groups. **** *p* < 0.0001.

Analyzing flies treated with SLAq, we found that, compared to their respective controls, the 2-week treatment with 1 mg/mL and 2.5 mg/mL SLAq improved performance in both males and females (Figure 2A,B).

Together, these data show that the treatment with SL extracts can provide resilience to age-related cognitive/locomotor decline, as determined by the fast phototaxis assay. While SLE had the most significant effects at 1 mg/mL, SLAq was equally efficient at 1 and 2.5 mg/mL. Although both extracts improved phototaxis, a comparison between SLE and SLAq showed that SLAq was more efficient than SLE (with the exception of females receiving the 1 mg/mL dose; Supplementary Table S2). In the case of SLAq, both 1 mg/mL and 2.5 mg/mL were equally protective in males and females (Supplementary Figure S1C,D).

### 2.2. Sleep Fragmentation Is Reduced in Males Given the Ethanol Extract of SL

Sleep fragmentation is characterized by frequent nighttime awakenings and subsequent daytime napping and is common during aging. In humans and flies, sleep disruptions have been shown to cause learning and memory impairment as well as overall cognitive decline and susceptibility to neurodegeneration [29]. Furthermore, fragmented sleep patterns are a common symptom in the elderly and have a severe impact on quality of life [3,34]. In order to determine whether a 2-week treatment with SLE or SLAq can reduce age-related sleep fragmentation, we assessed the number of sleep bouts per day and sleep bout duration in 6-week-old flies. At this age, flies show an increase in sleep bout number, while their duration is decreased when compared to younger flies [35].

**Figure 3:**
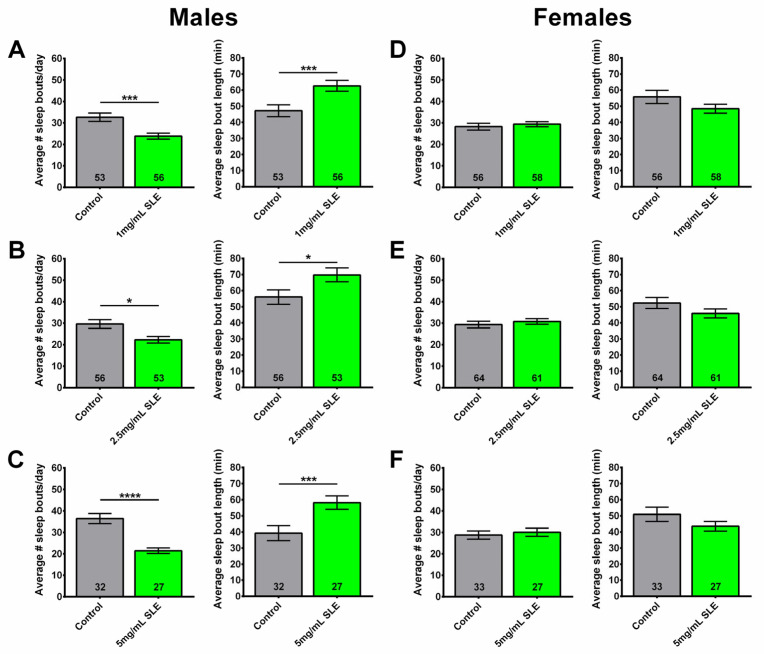
SLE treatment reduces sleep fragmentation in males. Treating 4-week-old male flies for two weeks with (**A**) 1 mg/mL, (**B**) 2.5 mg/mL, or (**C**) 5 mg/mL SLE reduced the number of sleep bouts (**left**), while increasing their length (**right**). (**D**–**F**) Sleep fragmentation in females was not reduced at any of the tested SLE concentrations. SLE data in green and gray for controls. Data are represented as the mean ± SEM of three independent experiments per treatment group. The total number of flies analyzed is denoted in each column. Due to the values not being normally distributed (D’Agostino–Pearson test), the non-parametric Mann–Whitney test was used to determine statistical significance between groups. * *p* < 0.05, *** *p* < 0.001, **** *p* < 0.0001.

At all concentrations (1, 2.5, and 5 mg/mL SLE), SLE-treated males had fewer sleep bouts per day, with longer average durations of sleep bouts compared to controls (Figure 3A–C). However, females given a 2-week treatment at 1, 2.5, and 5 mg/mL of SLE showed no significant effects on sleep fragmentation (Figure 3D–F). These data reveal that a 2-week SLE treatment in middle-aged male flies reduced sleep fragmentation compared to their respective age-matched controls, whereas it had no beneficial effect in females.

**Figure 4:**
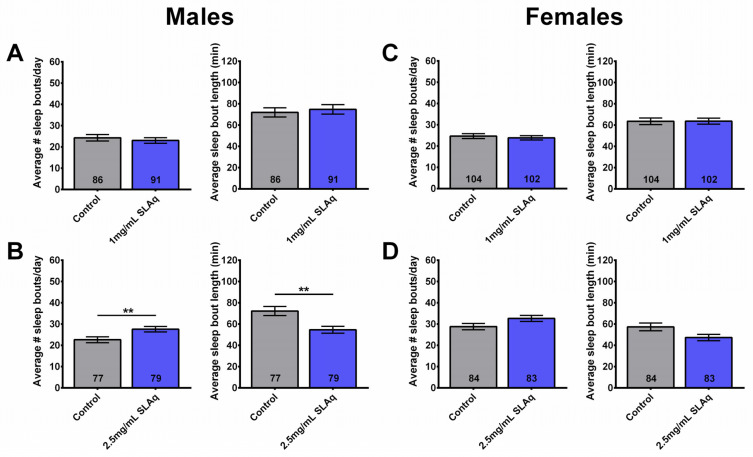
Two-week treatment with SLAq does not reduce sleep fragmentation. (**A**) Treatment of males with 1 mg/mL SLAq had no effect on sleep fragmentation, while treatment with (**B**) 2.5 mg/mL SLAq increased sleep fragmentation, as seen by the increase in sleep bout number per day (**left**) and the decrease in sleep bout length (**right**). (**C**,**D**) SLAq treatment at both concentrations had no effect on sleep fragmentation in females. SLAq data in blue and gray for controls. Data are represented as the mean ± SEM of three independent experiments per treatment group. The total number of flies analyzed is denoted in each column. Due to the values not being normally distributed (D’Agostino–Pearson test), the non-parametric Mann–Whitney test was used to determine statistical significance between groups. ** *p* < 0.01.

In contrast to the SLE treatment, no significant differences were observed in the number or duration of sleep bouts in males or females following a 2-week treatment with 1 mg/mL SLAq (Figure 4A,C). However, males treated with 2.5 mg/mL SLAq showed an increase in the number of sleep bouts per day, with the sleep bout duration being significantly shorter. There was no effect in females (Figure 4B,D). Similarly to SLE, 6-week-old females treated with SLAq at either concentration showed no significant effects on the duration or the number of sleep bouts per day compared to controls.

In summary, these results show that SLE supplementation reduces sleep fragmentation in 6-week-old males compared to controls. However, no improvement in sleep fragmentation was found in females treated with SLE. SLAq did not have a beneficial effect on sleep fragmentation in either females or males. Surprisingly, males treated with the higher dose of 2.5 mg/mL SLAq even showed increased fragmentation.

### 2.3. SLE Increased Nighttime Sleep

In addition to sleep fragmentation, we also assessed whether SL extract supplementation affects the amount of daytime, nighttime, and total amount of sleep.

**Figure 5:**
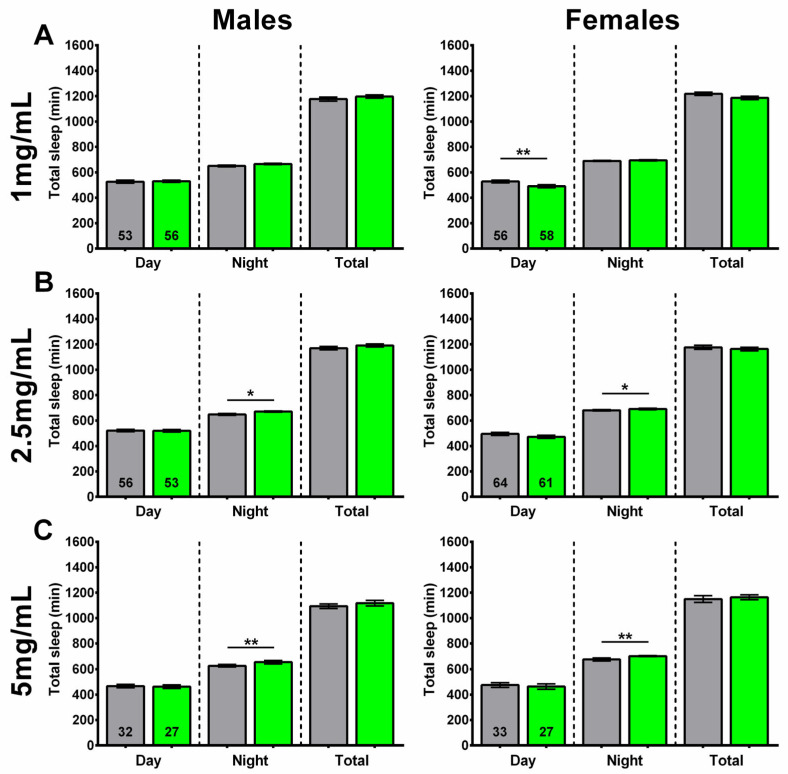
Higher concentrations of SLE increased nighttime sleep. (**A**) Supplementing the food of 4-week-old flies for 2 weeks with 1 mg/mL SLE did not affect the time male flies spend asleep (**left**), but it reduced daytime sleep in females (**right**). Giving SLE at (**B**) 2.5 mg/mL or (**C**) 5 mg/mL increased nighttime sleep in both males and females. SLE data in green and gray for controls. Data are represented as the mean ± SEM of three independent experiments per treatment group. The total number of flies analyzed is denoted in each column. Due to the values not being normally distributed (D’Agostino–Pearson test), the non-parametric Mann–Whitney test was used to determine statistical significance between groups. * *p* < 0.05, ** *p* < 0.01.

At the lower concentration of 1 mg/mL, SLE-treated females showed a decrease in daytime sleep, with no effect on nighttime or total sleep. No significant differences were seen in males treated with SLE at 1 mg/mL (Figure 5A). On the other hand, a treatment with 2.5 and 5 mg/mL SLE significantly increased the amount of nighttime sleep, with no effect on daytime or total sleep in both males and females (Figure 5B,C).

**Figure 6:**
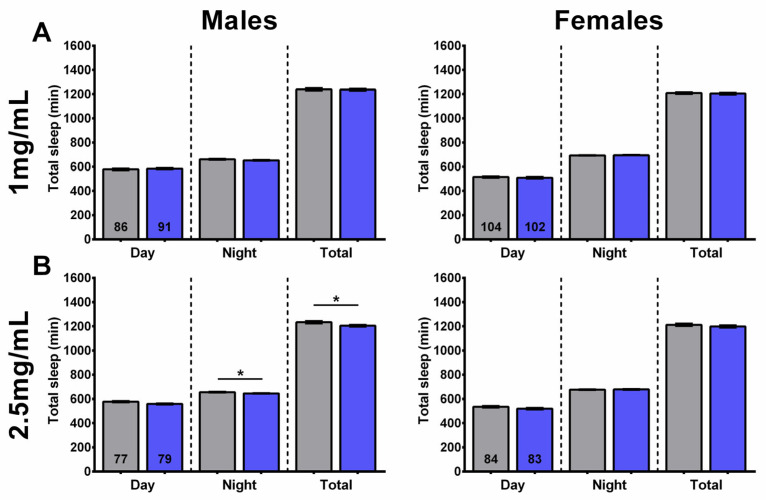
SLAq treatment did not increase sleep time. (**A**) Treatment with 1 mg/mL SLAq had no effect on the time spent asleep in either sex. (**B**) Treatment with 2.5 mg/mL SLAq reduced nighttime sleep and total sleep in males (**left**). It had no effect in females (**right**). SLAq data in blue and gray for controls. Data are represented as the mean ± SEM of three independent experiments per treatment group. The total number of flies analyzed is denoted in each column. Due to the values not being normally distributed (D’Agostino–Pearson test), the non-parametric Mann–Whitney test was used to determine statistical significance between groups. * *p* < 0.05.

In line with its effects on sleep fragmentation, the 1 mg/mL SLAq treatment had no significant effect on daytime, nighttime, or total sleep in either males or females compared to controls (Figure 6A). A lack of effect on daytime, nighttime, and total sleep was also evident in females treated with 2.5 mg/mL SLAq. However, males treated with 2.5 mg/mL SLAq showed a significant decrease in both nighttime and total sleep compared to controls (Figure 6B).

These findings show that, at higher concentrations, SLE treatment can increase nighttime sleep without a significant impact on daytime or total sleep in both sexes. At lower concentrations, SLE decreased the amount of daytime sleep in females but had no effect in males. In contrast, the SLAq treatment had no effect on the amount or timing of sleep in females; however, it reduced nighttime and total sleep in males at the higher concentration of 2.5 mg/mL.

### 2.4. SL Supplementation Did Not Extend Longevity

To determine whether resilience to age-related functional decline is accompanied by delayed aging, we also assessed mortality in SLE- and SLAq-treated and control flies. For these experiments, treatment began at 4 weeks of age, and the flies were maintained on their respective diet throughout the remainder of their lives. Although males and females were housed together, mortality was analyzed separately in three independent experiments for each extract type and concentration, alongside controls.

**Figure 7:**
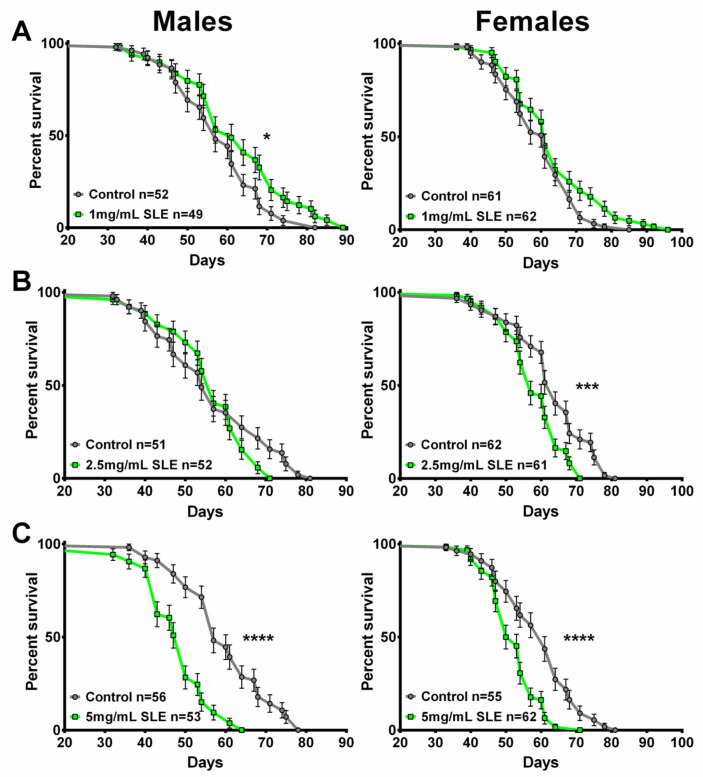
SLE treatment does not extend lifespan. (**A**) SLE at 1 mg/mL (green) had no effect on lifespan in females and slightly increased it in males. (**B**) Treatment with 2.5 mg/mL SLE had no effect in males, whereas in females the lifespan was decreased. (**C**) At the highest concentration of 5 mg/mL, lifespan was decreased in both sexes. Data are represented as the mean ± SEM from three independent experiments per treatment group. Statistical significance was determined using Log-rank (Mantel–Cox) tests. Total n is indicated in the figures; n per test is listed in Supplementary Table S3. * *p* < 0.05, *** *p* < 0.001, **** *p* < 0.0001.

**Figure 8:**
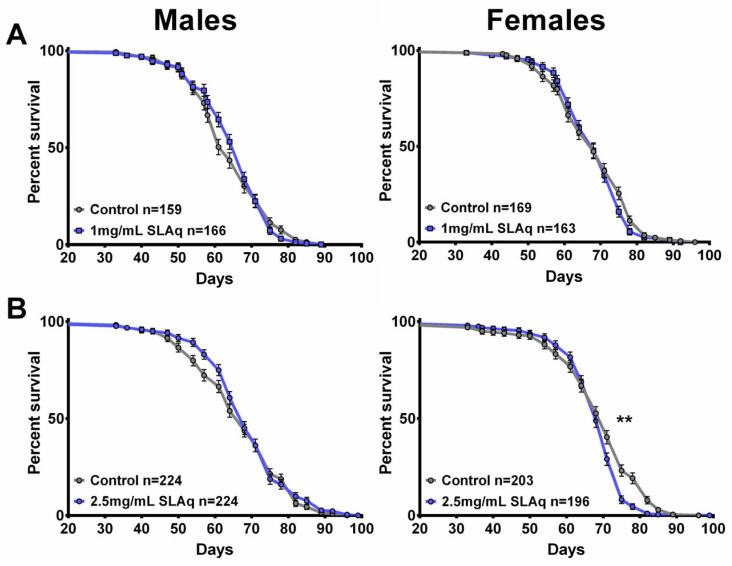
SLAq does not improve lifespan. (**A**) Compared to their respective controls, 1 mg/mL SLAq (blue) treatments had no significant effect on lifespan. (**B**) Treatment with 2.5 mg/mL had no effect in males and reduced lifespan in females. Data are represented as the mean ± SEM from three independent experiments per treatment group. Statistical significance was determined using Log-rank (Mantel–Cox) tests. Total n is indicated in the figures; n per test is listed in Supplementary Table S3. ** *p* < 0.01.

The treatment with the lower dose of SLE at 1 mg/mL did not extend lifespan in females but slightly improved survival at later stages in males compared to controls (Figure 7A). In contrast, females show a reduction in survival with 2.5 mg/mL SLE, whereas males show no significant effect (Figure 7B). With SLE at 5 mg/mL, a reduced lifespan was detected in both females and males (Figure 7C). In the case of SLAq treatment, neither 1 mg/mL nor 2.5 mg/mL had a significant effect on lifespan, with the exception of 2.5 mg/mL in females, which showed a slight reduction (Figure 8A,B). These data suggest that SL supplementation does not provide resilience to age-related phenotypes by extending the median lifespan but may have more direct effects on these phenotypes. This is also supported by the findings that SLE can improve phototaxis and sleep, whereas SLAq has no effect on sleep but does improve phototaxis.

### 2.5. SLE Reduces Neurodegeneration

**Figure 9:**
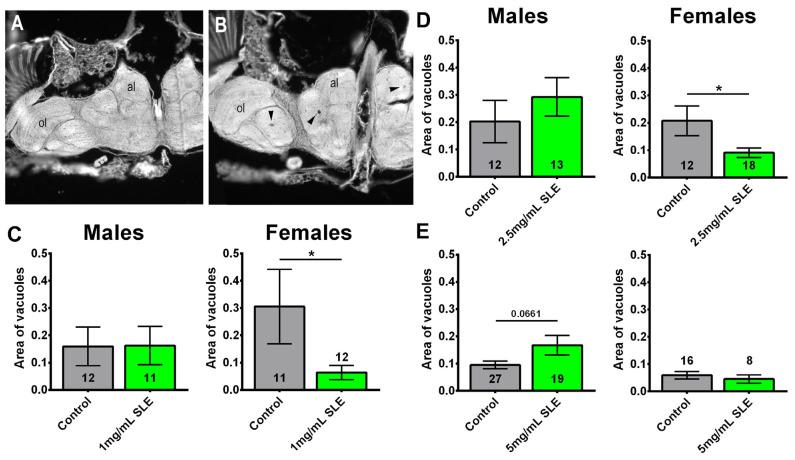
SLE reduces degeneration in females. (**A**) Head section showing a fly treated with 1 mg/mL that does not show vacuolization. (**B**) Head section from a control that shows several vacuoles (arrowheads). al = antennal lobes, ol = optic lobes. Females treated with (**C**) 1 mg/mL or (**D**) 2.5 mg/mL SLE show less degeneration, as measured by the area of vacuoles in the brain. Treatment with (**E**) 5 mg/mL has no effect. There is no effect in males at any of the concentrations. Controls are shown in gray; SLE is shown in green. Data are represented as the mean ± SEM. The total number of flies analyzed is denoted in each column. Due to the values not being normally distributed (D’Agostino–Pearson test), the non-parametric Mann–Whitney test was used to determine statistical significance between groups. * *p* < 0.05.

As the SLE treatment did not significantly affect the median lifespan but improved cognitive/locomotor performance and/or reduced sleep fragmentation, we sought to determine whether resilience to these age-related phenotypes is due to protection of the brain. To determine this, we measured the area of vacuoles in the brains of 8-week-old flies fed an SLE or control diet starting at 4 weeks of age. Flies were analyzed at eight weeks of age due to degeneration being detectable at this age in CS [36] and Figure 9B. We focused on SLE because it improved phototaxis and sleep. The treatment with 1 mg/mL reduced neurodegeneration in females (Figure 9A,C) but not in males (Figure 9C). This effect was also observed in females treated with 2.5 mg/mL SLE; however, treated males were not significantly different from controls (Figure 9D). Although we also detected a reduction in the degeneration in females treated with 5 mg/mL, this did not reach significance. Males treated with 5 mg/mL SLE showed an increase in degeneration (Figure 9E). The reduction in age-related neurodegeneration could contribute to improvements in some behavioral deficits.

### 2.6. LC-MS Identification and Quantification of 10 Flavonoids in SLE and SLAq

Several studies have reported the chemical constituents of *Scutellaria* species and their extracts. Flavonoids are the major constituents of *Scutellaria* species, although the quantities that are present in different tissues differ between the species. In order to identify the possible active compounds that mediate the beneficial effects of our SL extracts, we set out to quantify 10 flavonoids, namely baicalin, baicalein, wogonin, wogonoside, norwogonin, chrysin, apigenin, oroxylin A, scutellarin, and scutellarein, in the extracts. Of the eight flavonoids quantified in SLE, only three were also found in SLAq (see Table 1). The three compounds detected in both extracts were baicalin, wogonoside, and oroxylin A, with higher levels found in SLE compared to SLAq. Of the compounds quantified, baicalin was by far the most abundant, with a concentration of 97.5358 µg/mg in SLE and 91.9381 µg/mg in the SLAq extract. The next most abundant compound in both the SLE and SLAq extracts was wogonoside, at 1.1236 and 0.8678 µg/mg, respectively. Scutellarein was the third most abundant flavonoid, with a concentration of 0.0110 µg/mg in our SLE, but it was not detected in SLAq. The concentrations of all tested compounds per mg of SLE and SLAq extract are shown in Table 1.

Three different compound diets were made: one containing baicalin alone, one containing wogonin, wogonoside, and norwogonin (wogon mix), and lastly one containing apigenin and scutellarein (AS mix). The baicalin stock solution was made by dissolving baicalin in EtOH due to its low solubility in DMSO or water. Instead of creating a diet that only contains wogonoside, we decided to include two of its aglycones, wogonin and norwogonin. Both of these compounds were detected and quantified in SLE but not in SLAq. These compounds were dissolved in DMSO; the control stock, therefore, contained the same concentration of DMSO. All three of these compounds have been reported to have anti-inflammatory, antioxidant, anti-viral, and anticancer properties [37]. Apigenin is a widely abundant flavonoid found in various plants [38]. Scutellarein is a derivative of apigenin, and both were present in SLE but not SLAq. Apigenin and scutellarein were also dissolved in DMSO to make the AS mix stock. Among others, antioxidant and anti-inflammatory properties have been reported for apigenin [38] and scutellarein [39].

As SLE at 1 mg/mL was effective in improving cognitive/locomotor performance and sleep fragmentation (in males), select compounds were used in quantities equivalent to 1 mg/mL SLE. Control diets were made for each compound diet containing the equivalent amount of solvent. As we did with the SL extracts, 4-week-old flies were fed one of the compound diets or its respective control diet for 2 weeks, then assessed for age-related phenotypes.

### 2.7. Compound Supplementation Improved Performance in Phototaxis

**Figure 10:**
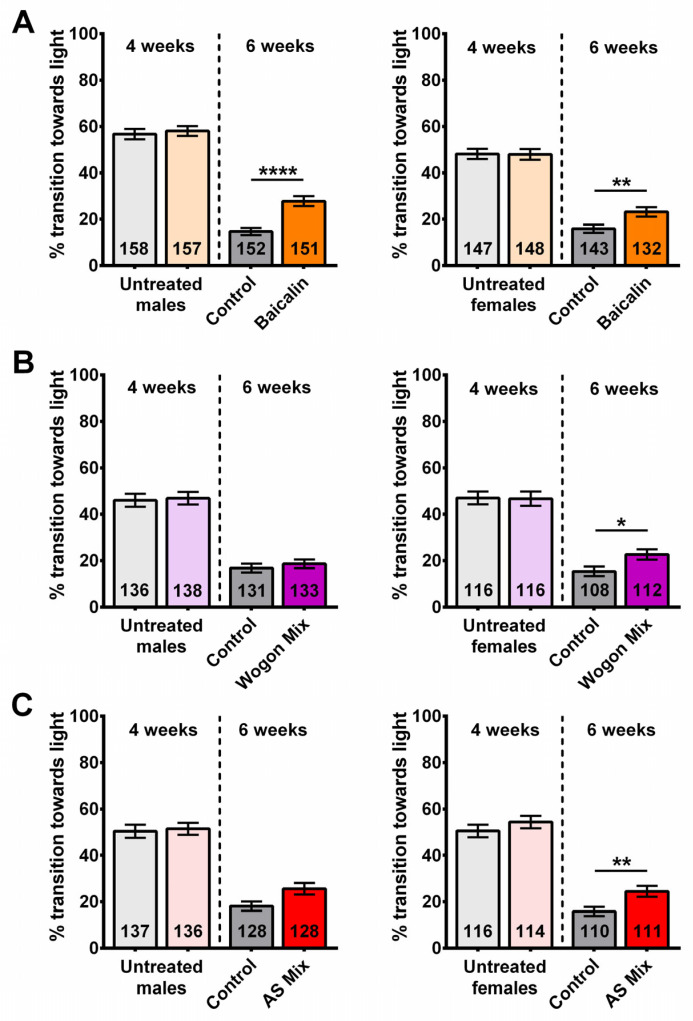
A 2-week treatment with a baicalin-supplemented diet improves cognitive/locomotor performance in 6-week-old flies. Performance in phototaxis declines between 4 weeks and 6 weeks of age. (**A**) Six-week-old baicalin-treated (orange) flies performed better than age-matched controls (gray) in both males (**left**) and females (**right**). Two-week treatment with (**B**) wogon mix (purple) and (**C**) AS mix (red) improved phototaxis in females (**right**) but not significantly in males (**left**). Data are represented as the mean ± SEM of three independent experiments per treatment group. The total number of flies analyzed is denoted in each column. Due to the values not being normally distributed (D’Agostino–Pearson test), the non-parametric Mann–Whitney test was used to determine statistical significance between groups. * *p* < 0.05, ** *p* < 0.01, **** *p* < 0.0001.

Using the same protocol as for the SL extracts, we tested male and female flies separately before treatment at 4 weeks of age and after the following 2 weeks of treatment at 6 weeks of age. Prior to treatment, 4-week-old flies showed no significant differences in cognitive/locomotor performance between groups in males or females (Figure 10A–C). At six weeks of age, flies that received a baicalin-supplemented diet showed a significantly improved performance in males and females compared to the controls (Figure 10A). An improved performance in the phototaxis assay was also observed in wogon mix-fed females compared to controls; however, there was no significant difference between treated and control males (Figure 10B). Similarly, female flies on the AS-supplemented diet performed significantly better than controls, whereas in males there was a positive trend that did not reach significance (Figure 10C). Two-way ANOVA analyses are shown in Supplementary Table S1.

Combined with the SL extract results, this suggests that baicalin plays a major role in providing resilience against age-related cognitive/locomotor decline, as it is present in SLE and SLAq and improves behavior in both male and female flies. However, the wogonoside contained in the wogon mix, which is also present in SLE and SLAq, might contribute to the improvement in females. Neither apigenin nor scutellarein was detected in our SLAq extract, and we therefore assume they play only a minor role, although they may contribute to the effects of SLE.

### 2.8. Wogon Mix Treatment Reduced Sleep Fragmentation in Males

To investigate which compounds may contribute to the reduced sleep fragmentation seen in male flies treated with SLE (1 mg/mL), the number of sleep bouts and their average duration were assessed in 6-week-old flies over 3 days.

**Figure 11:**
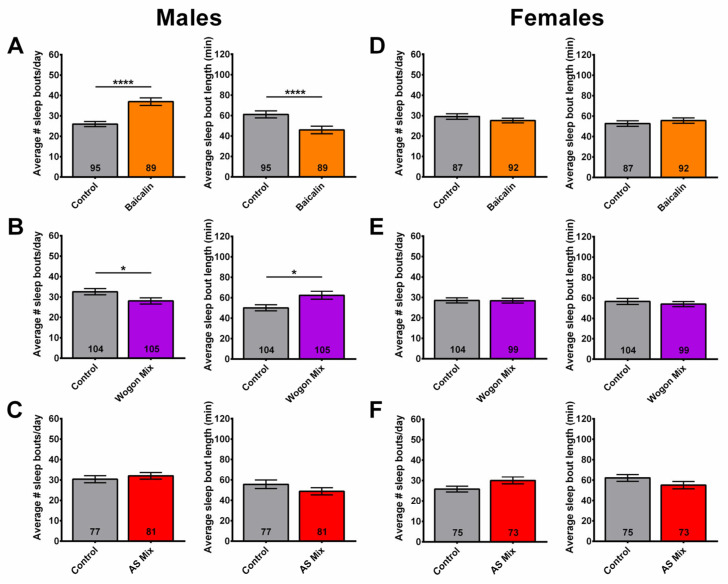
Wogon mix treatment reduces age-related sleep fragmentation in males. (**A**) Compared to age-matched controls (gray), the number of sleep bouts per day increased and the duration decreased in baicalin-treated (orange) males. (**B**) Wogon mix-treated (purple) males had fewer sleep bouts per day of significantly longer duration compared to controls. (**C**) AS mix (red) treatment had no effect on sleep fragmentation in males. Compared to control females (gray), two-week treatment with (**D**) baicalin (orange), (**E**) wogon mix (purple), or (**F**) AS mix (red) had no significant effect on sleep fragmentation. Data are represented as the mean ± SEM of three independent experiments per treatment group. The total number of flies analyzed is denoted in each column. Due to the values not being normally distributed (D’Agostino–Pearson test), the non-parametric Mann–Whitney test was used to determine statistical significance between groups. * *p* < 0.05, **** *p* < 0.0001.

Consistent with the sex-specific effects observed with SLE supplementation, neither baicalin nor the two mixes improved sleep fragmentation in females (Figure 11D–F). Males treated with baicalin had more sleep bouts per day of significantly shorter duration compared to controls (Figure 11A), similar to what we observed after SLAq treatment at the higher concentration. In contrast, the wogon-supplemented males had fewer sleep bouts per day of significantly longer duration (Figure 11B). Since wogonin and norwogonin, in contrast to wogonoside, are only present in SLE, either or both of these compounds may mediate the function of SLE in improving sleep fragmentation in males. The similarity in the number and duration of sleep bouts in AS mix-treated males to those in controls (Figure 11C) suggests that neither apigenin nor scutellarein plays a significant role in providing resilience to age-related sleep fragmentation.

### 2.9. Wogon Mix Treatment Has No Effect on Sleep Timing

**Figure 12:**
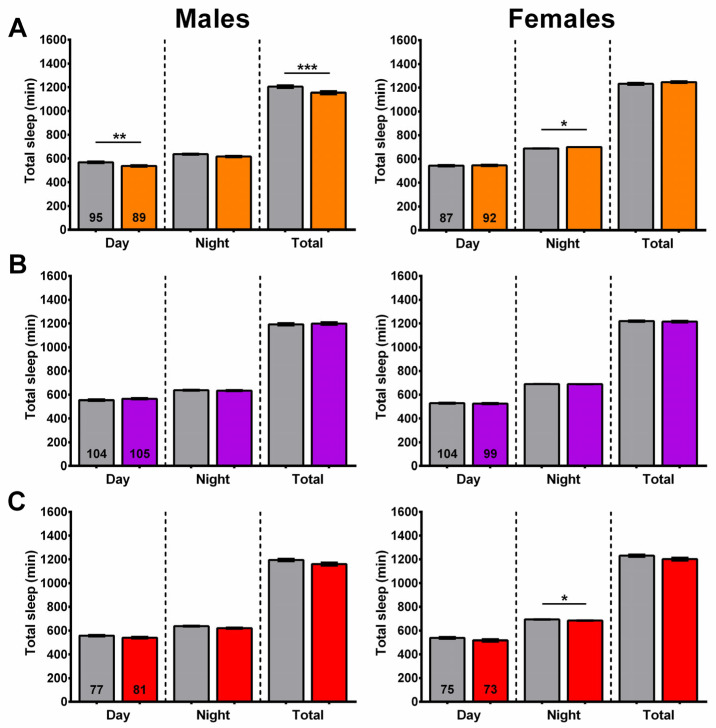
Sleep time is only minimally affected by compound treatment. (**A**) Baicalin supplementation (orange) reduces daytime sleep and total sleep in males and slightly increased nighttime sleep in females. (**B**) The wogon mix (purple) had no significant effect on sleep timing in males or females. (**C**) AS mix (red) slightly decreased nighttime sleep in females with no effect in males. Data are represented as the mean ± SEM of three independent experiments per treatment group. The total number of flies analyzed is denoted in each column. Due to the values not being normally distributed (D’Agostino–Pearson test), the non-parametric Mann–Whitney test was used to determine statistical significance between groups. * *p* < 0.05, ** *p* < 0.01, *** *p* < 0.001.

In addition to sleep fragmentation, we also assessed the amounts of daytime, nighttime, and total sleep in flies treated with baicalin, wogon mix, and AS mix diets. Compared to controls, 6-week-old baicalin-treated males had a significant decrease in daytime and total sleep, while nighttime sleep was increased in females (Figure 12A). An increase in nighttime sleep was also observed after SLE treatment, although this effect was only evident at the 2.5 and 5 mg/mL concentrations and was detected in both males and females. The 2-week treatment with the wogon mix had no effect on daytime, nighttime, or total sleep in either males or females (Figure 12B). The AS mix supplementation also had no effect on sleep timing in males; however, in females, there was a slight but significant decrease in nighttime sleep (Figure 12C).

Overall, the effects on sleep timing varied across treatments, and in some treatments they were sex-specific. The baicalin treatment had different effects on sleep timing, with males sleeping less during the day and females spending more time asleep at night. While the wogon mix supplementation reduced sleep fragmentation in males, it had no significant effect on sleep timing in either males or females. The AS-supplemented diet had no effect on sleep fragmentation in males or females; however, it did decrease the amount of time spent asleep at night in females.

### 2.10. Longevity

**Figure 13:**
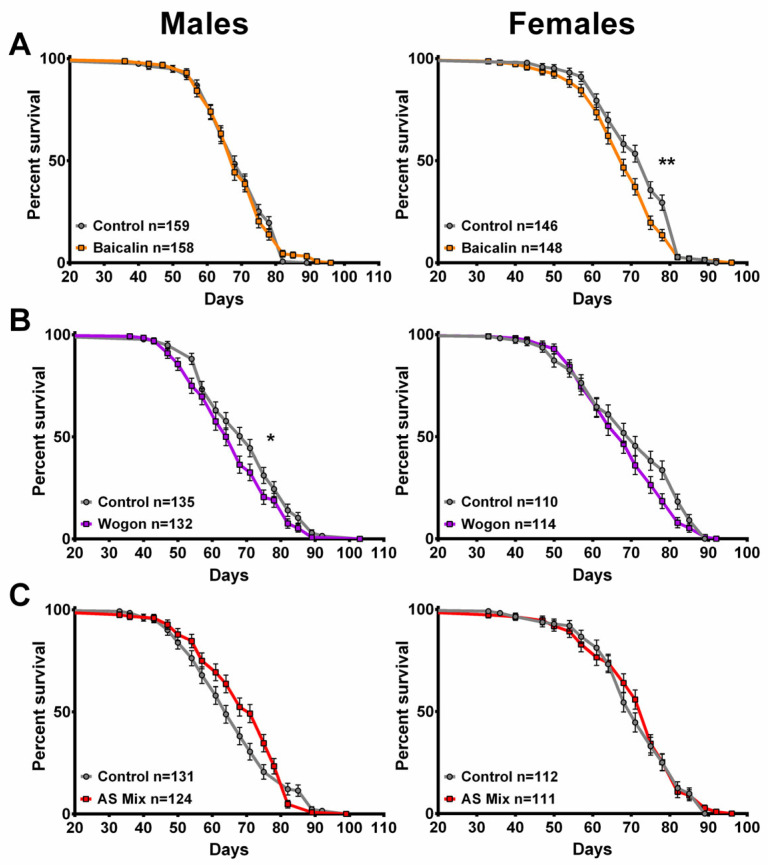
None of the compounds improved lifespan. (**A**) Compared to their respective controls, baicalin (orange) had no significant effect on males, but it decreased survival in females. (**B**) Wogon mix (purple) slightly decreased survival in males and had no effect in females. (**C**) The AS mix had no effect on lifespan, neither in males nor females. Data are represented as the mean ± SEM from three independent experiments per treatment group. Statistical significance was determined using Log-rank (Mantel–Cox) tests. Total n is indicated in the figures; n per test is listed in Supplementary Table S3. * *p* < 0.05, ** *p* < 0.01.

Similarly to the results of SLE and SLAq supplementation, there were only small differences in the lifespan of flies treated with any of the compound diets. There was a slight reduction in the lifespan of baicalin-treated females and in wogon mix-treated males, whereas the other treatments had no effect (Figure 13A–C).

### 2.11. The Neurodegeneration Was Not Improved by the Tested Compounds

**Figure 14:**
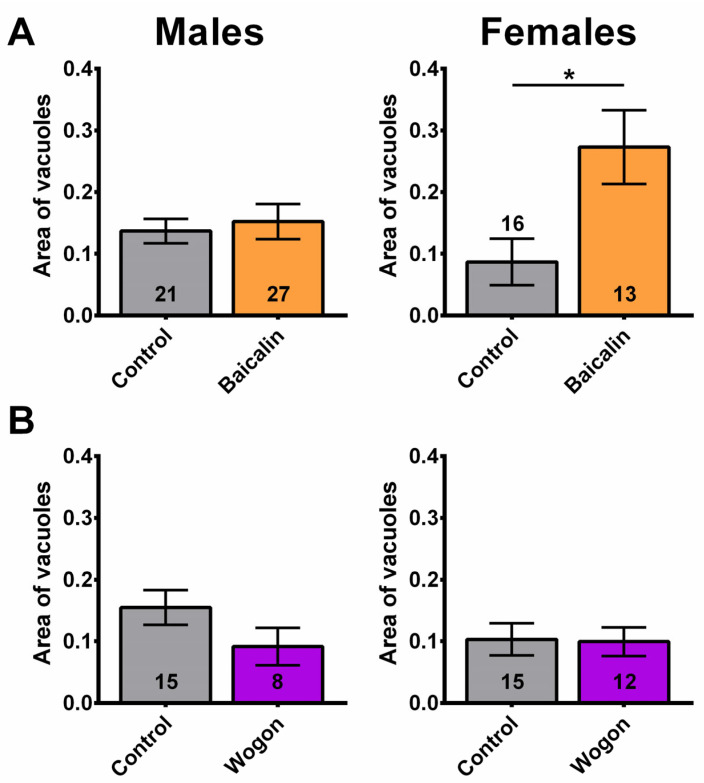
The compounds did not improve neurodegeneration. (**A**) While males are unaffected by baicalin supplementation (orange), females show increased neurodegeneration. (**B**) The wogon mix (purple) had no significant effect in females or males compared to controls (gray). Data are represented as the mean ± SEM. The total number of flies analyzed is denoted in each column. Due to the values not being normally distributed (D’Agostino–Pearson test), the non-parametric Mann–Whitney test was used to determine statistical significance between groups. * *p* < 0.05.

Lastly, we tested the effects of the compounds on neurodegeneration. Due to the AS mix not having an effect on behavior, the AS mix was not included. Using baicalin, we detected no effect on age-related neurodegeneration in males, but in females the degeneration was increased (Figure 14A). By treating with the wogon mix, we detected a decrease in the degeneration in males, but this did not reach significance (Figure 14B). It had no effect in females.

## 3. Discussion

As mentioned in the introduction, *S. lateriflora* has been used in herbal medicine for centuries for its sedative, anxiolytic, and pain-relieving effects [27,40].

SL has also been used in studies in rodents. In rodents, it has been shown to reduce anxiety levels, and it has been suggested that baicalin and baicalein act as the neuroactive flavonoids interacting with the γ-aminobutyric acid-A (GABA_A_) receptor complex [37,41,42,43]. Mice given SL as a tea continuously starting 2 weeks prior to inoculation with scrapie (prion disease) survived significantly longer than controls. SL tea, baicalin, and baicalein inhibited fibril formation and destabilized formed fibrils in vitro [44].

Whether SL extracts can provide resilience to age-related impairments has, to our knowledge, so far not been addressed in animal models or clinical trials. We now studied this in the *Drosophila* model, aging them for four weeks to reach middle age before treating them with extracts made from the aerial parts of *S. lateriflora*. We first tested the effects on age-related declines in mobility and reaction, using the fast phototaxis assay. As expected, the performance in the fast phototaxis assay declined with age in all flies tested. However, the 2-week treatment with 1 mg/mL SLE improved locomotor/reaction performance in both males and females. At higher SLE concentrations, the treated flies also performed better than the controls; however, this improvement did not always reach significance in both sexes. SLAq had a more robust effect on phototaxis because both males and females performed better after treatment with 1 and 2.5 mg/mL SLAq. Testing the compounds, we also found a robust improvement in phototaxis with baicalin. This shows that baicalin is sufficient for improving phototaxis, and, due to baicalin being present in both extracts, this suggests that this compound is mediating the beneficial effects of both SLE and SLAq on cognitive/locomotor performance. Although *S. lateriflora* has not been tested for an improvement in mobility or reaction, a herbal supplement containing another *Scutellaria* species, *S. baicalensis*, as well as *C. laevigata* and magnesium/chromium, did improve recognition reaction time and cognition in healthy but stressed subjects aged 18–75 [45]. This publication also describes that the study participants reported improved sleep, although this was only a subjective assessment. Sleep disruptions are a common occurrence in the elderly, with problems falling asleep and waking up frequently during the night [3,4,5]. Similarly, *Drosophila* show a more interrupted sleep pattern, detectable through an increase in sleep bout number and a decrease in their length [35].

We therefore determined the sleep pattern in SL-treated flies as a second measurement for effects on age-related changes. In contrast to the phototaxis tests, SLAq had either no effect on the sleep patterns or even increased sleep fragmentation in males at the higher dose of 2.5 mg/mL. However, SLE reduced sleep fragmentation at all three concentrations tested, although only in males. At the higher concentrations of 2.5 mg/mL and 5 mg/mL, it also significantly increased the total time of nighttime sleep, and, surprisingly, this was the case in both males and females. This shows that the effects of SL on sleep are dependent on the preparation of the extract, suggesting that SLE contains compounds that are not, or are not efficiently, extracted by water. Lohani and colleagues reported that 70% EtOH extracts of *S. lateriflora* contained higher levels of protein, glutathione, polyphenols, and flavonoids compared to aqueous extracts [19]. This was confirmed by our analyses, which showed that only three of the eight flavonoids found in SLE were also present in SLAq, and even those were less concentrated in SLAq compared to SLE. When testing whether the effects of SLE on sleep are mediated by one or some of these flavonoids, we found that baicalin actually increased sleep fragmentation in males at a concentration corresponding to 1 mg/mL SLE. In contrast, the mix of wogonin, wogonoside, and norwogonin reduced sleep fragmentation in males. That baicalin did not improve sleep is consistent with SLAq not improving sleep, as both SLE and SLAq contain significant amounts of baicalin. The fact that we do not detect a negative effect on sleep with the SLAq extracts suggests that other compounds in the extracts might overcome the negative effect of baicalin on sleep. Whereas wogonoside is found in both extracts, wogonin and norwogonin were only detected in SLE, and we therefore assume that these two compounds play an important role in improving sleep fragmentation. Using rats, it was shown that baicalin has complex effects on sleep, decreasing slow wave sleep during the light period and increasing it during darkness, and these effects are mediated by GABA_A_ [46]. As mentioned above, the activation of GABA_A_ receptors was also suggested to account for the anxiolytic effect of baicalin in mice [37]. The effects of wogonin or norwogonin on sleep have, to our knowledge, not been tested in other models.

As when testing SLE, the effect of the compounds on sleep was restricted to males, showing that the preparation and concentration of specific compounds is important but also that the functional mechanism of these compounds might be different in males and females. This is in contrast to the phototaxis, which is improved in both males and females. Lastly, we tested whether the improvements in age-related cognitive/locomotor performance and sleep deficits in our *Drosophila* model are due to SL slowing the aging process. However, neither SLE, SLAq, nor any of the compounds extended lifespan, and in some cases they even reduced survival, and it is therefore unlikely that the improvement is caused by slowing the aging process. However, we found that SLE reduced neurodegeneration in females, suggesting that SL can maintain brain health during aging and thereby improve neuronal function and behavior. As with sleep, baicalin had a negative effect on neurodegeneration, again suggesting that the negative effects of baicalin are mitigated by other compounds in the SLE mix. In contrast to baicalin, the wogon mix reduced sleep fragmentation in males, and, although it did not quite reach significance, it also reduced neurodegeneration in males. It is therefore possible that wogonin and/or norwogonin improve sleep by promoting the health of the neurons regulating sleep.

## 4. Materials and Methods

### 4.1. Drosophila Melanogaster Stocks

*Drosophila melanogaster* (Canton S) were maintained under a 12 h:12 h light/dark (12:12 LD) cycle at 25 °C. Adult male and female wildtype (Canton S) flies were maintained on a *Drosophila* diet (agar: 338 g, cornmeal: 2149 g, yeast: 696.5 g, sugar: 425 g, old inhibitor: 100 g, ethanol: 348 mL, diH_2_O: 36 L) until four weeks of age and then transferred to fresh food with the extracts/compounds or vehicle. Fresh diet was provided every 3–4 days.

### 4.2. Raw Plant Material and Extracts

*Scutellaria lateriflora* dried aerial parts were purchased through Oregon’s Wild Harvest (OWH), Redmond, OR, USA (OWH Lot# 231000203). A 70% ethanol extract (SLE) and an aqueous extract (SLAq) were prepared as described by [31]. Briefly, the 70% ethanol extract was prepared by boiling the dried aerial parts under reflux with 70% ethanol in water for 90 min at a ratio of 80 g of material to 1 L of 70% ethanol. To remove larger plant fragments, the warm extract was filtered through a sieve and then centrifuged (Beckman GS-6R) at 3850 rpm for 10 min to sediment finer particles. The pellet containing plant particles was discarded, and the supernatant was filtered through Whatman filter paper (Grade 1, 90 mm). Ethanol was then removed from the filtrate under vacuum on a rotary evaporator, and the remaining predominantly aqueous solution was frozen and lyophilized into a powder on a Virtis lyophilizer (Phase 1, 115 V, 20 amps). The yield of dried 70% ethanol extract was about 23.9% by weight of the original plant material. The aqueous extract was prepared by boiling *S. lateriflora* dried aerial parts in deionized water under reflux for 90 min at a ratio of 160 g of material to 2 L of water. The mixture was then filtered through a sieve and centrifuged, and the supernatant was filtered through filter paper as described for the ethanol plant extract above. This extract was then frozen and lyophilized into a powder as described for the ethanol extract. The yield of dried water extract was about 15.1% by weight of the original plant material. Each extraction was given a specific lot number, BEN-SLE-1 (ethanol extract) and BEN-SLAq-1 (aqueous extract), and stored at −20 °C until use. A voucher specimen of the dried plant material is stored at the BENFRA laboratory at OHSU under the code number BEN-SL-1.

### 4.3. Chemicals and Reagents

Organic solvents (HPLC grade) and general laboratory supplies such as vials and pipette tips were obtained from Fisher Scientific (Fairlawn, NJ, USA) or Avantor (Allentown, PA, USA). Reference standards for oroxylin A, norwogonin, and scutellarein were obtained from Cayman Chemical (Ann Arbor, MI, USA); wogonoside was obtained from LGC Standards (Manchester, NH, USA); and Chromadex (Longmont, CO, USA) supplied the Skullcap standards kit, which included baicalin, baicalein, scutellarin, and wogonin. Chrysin (Chromadex) and apigenin (Sigma, St. Louis, MO, USA) were kindly provided by Dr. Amala Soumyanath.

### 4.4. Preparation of Scutellaria lateriflora Extracts and LC-MS/MS Method Calibrators

Lyophilized *Scutellaria lateriflora* extracts were reconstituted in water to a concentration of 50 mg/mL. Extracts were then serially diluted with water to 5 mg/mL, 2.5 mg/mL, 1 mg/mL, and 0.5 mg/mL. During analysis, the 0.5 mg/mL extracts were further diluted 1:10 in series to 0.05, 0.005, and 0.0005 mg/mL for the analysis of specific analytes with higher intensities (baicalin and scutellarin). An aliquot of the ethanol and water extract 1 mg/mL reconstituted stock was spiked with reference standard solution to confirm analyte retention time and evaluate potential matrix effects. Where possible (wogonin, chrysin, oroxylin A, norwogonin, apigenin, and scutellarein), the recovery of known amounts of reference standard spike was assessed and was satisfactory (within ±30%). Reference standard solutions were made in DMSO at a 1 mg/mL concentration. Curves were prepared for reference standards by diluting DMSO stocks in water to concentrations ranging from 1 ng/mL to 5000 ng/mL (scutellarein, wogonoside, baicalin, baicalein, and apigenin), or from 1 ng/mL to 1000 ng/mL (wogonin, chrysin, oroxylin A, norwogonin, and scutellarin). Dilution linearity was satisfactory except for higher-concentration compounds, where saturation occurred.

### 4.5. LC-MS/MS Analysis for S. lateriflora Extracts

*S. lateriflora* extracts were analyzed using a 5500+ QTRAP hybrid/triple quadrupole linear ion trap mass spectrometer (Applied Biosystems, Foster City, CA, USA) with electrospray ionization (ESI) in positive mode. The mass spectrometer was interfaced to a Shimadzu HPLC (Columbia, MD, USA) with an SIL-20AC XR auto-sampler, followed by two LC-20AD XR LC pumps. The mass spectrometer was operated with the following settings: source voltage 4500 kV, GS1 30, GS2 70, CUR 35, TEM 700, and CAD gas MED. Compounds were infused individually, and tandem mass spectrometry (MS/MS) instrument collision energy parameters were optimized for each parent to produce ion Multiple Reaction Monitoring (MRM) transitions. The scheduled MRM transitions used for quantification are provided in Table 2 and were monitored with a 25 ms dwell time. Where possible, a second transition was also monitored as a qualifying transition to improve method specificity. The HPLC gradient mobile phase was delivered at a flow rate of 0.3 mL/min and consisted of two solvents: A: 0.1% formic 10 mM ammonium formate in water and B: 0.1% formic in acetonitrile. The initial solvent B percentage was 5%, which was held for 0.5 min, followed by a 1 min gradient to 30%, and then an 8.5 min gradient to 60%, followed by a quick ramp of 0.5 min from 60% to 95%, held at 95% for 1 min to wash, 0.1 min to start, and re-equilibrated till 15 min. The injection volume was 5 µL and the flow was to the source, between 1.5 and 7 min. The LC column used was a Kinetex Phenyl-Hexyl 50 × 2.1 mm 2.6 µM, and the column oven was set to 40 °C; the autosampler was set to 15 °C, with a needle rinse before and after each injection. Data were acquired using Analyst 1.7.3 and analyzed with Multiquant 3.03.

### 4.6. Diets

Stock solutions were prepared by dissolving the lyophilized ethanol and aqueous extracts in double distilled water to a concentration of 50 mg/mL. Ethanol and aqueous stock solutions were diluted in the *Drosophila* diet at multiple final test concentrations, hereafter referred to as SLE and SLAq, respectively. Wogon mix and AS mix stocks were made by dissolving commercially available pure powdered compounds in DMSO, while baicalin was dissolved in EtOH. Each of the tested mixes contained the specified compounds (Table 1) at concentrations equivalent to those found per mg of SLE. Wogon mix, AS mix, and baicalin stock solutions were mixed into the *Drosophila* diet equivalent to 1 mg/mL SLE. Control diets were made by adding the equivalent amounts of the respective solvents (H_2_O, DMSO, or EtOH) into the *Drosophila* diet.

### 4.7. Fast Phototaxis

At 4 weeks of age, male and female flies were anesthetized and separated and allowed to recover for 12–16 h. Their cognitive/locomotor performance was then measured using the fast phototaxis assay as described previously [47]. After a two-week treatment with either a supplemented or control diet, six-week-old male and female flies were retested as described in [47]. Briefly, after 4 h of starvation, groups of up to 25 flies were placed in the concurrent apparatus described by [48]. In the dark, with a single light source, each experiment consisted of five successive tests of 6 s to transition into the next vial towards the light. At the end of the experiment, based on which of the six vials they reached, a score was given for each fly. Using GraphPad Prism (GraphPad Prism v6.07; GraphPad Software Inc., San Diego, CA, USA) and the D’Agostino–Pearson test, we found that the data were non-parametric, and therefore statistical significance between groups was determined using the Mann–Whitney test.

### 4.8. Sleep

At four weeks of age, the flies were switched to either a supplemented diet or their respective control diet. After two weeks of treatment with a supplemented or control diet, six-week-old individual flies were placed into individual tubes containing the specified diet, with one end sealed with paraffin and a piece of yarn at the other. Tubes were loaded into the TriKinetics *Drosophila* Activity Monitoring system (DAMS; TriKinetics, Waltham, MA, USA) and allowed to acclimate for 12–16 h. Data were collected every minute for 3 days under 12:12 LD at 25 °C. Sleep was defined as 5 or more consecutive minutes without a beam break [49,50]. The number of sleep bouts per day, sleep bout duration, and daytime, nighttime, and total sleep data were analyzed using ClockLab software (v6.1.02 Actimetrics, Lafayette Instrument Company, Lafayette, IN, USA). For statistical analysis, treated male and female flies were compared with sex-matched flies fed the respective control diet using the D’Agostino–Pearson test and the non-parametric Mann–Whitney test (GraphPad Prism v6.07; GraphPad Software Inc., San Diego, CA, USA).

### 4.9. Lifespan

At four weeks of age, the flies were switched to either a supplemented diet or their respective control diet. Mortality was recorded every 3–4 days at diet exchange without anesthesia. Sex was determined after death. Statistical significance of the median lifespan from three cohorts each between sex-matched flies on the supplemented diets and the respective control diets was determined through one-tailed unpaired *t*-tests with Welch’s correction using GraphPad Prism 6 (GraphPad Prism v6.07; GraphPad Software Inc., San Diego, CA, USA).

### 4.10. Neurodegeneration

At four weeks of age, flies were switched to either a supplemented diet or their respective control diet for four weeks. At eight weeks of age, the heads of male and female flies were randomly placed side by side in a collar and processed as a single sample, as previously described in [51]. Eight-week-old flies were analyzed because degeneration was detectable at this age in CS [36]. Briefly, heads were embedded in paraffin blocks, cut into 7 µm serial sections, and SafeClear (Fisher Scientific) was used to remove the paraffin. The sections were then embedded in Permount. Using an Olympus BX61 microscope (Olympus, Tokyo, Japan), sections were analyzed by an experimenter blinded to the treatments. The area of vacuoles, representing a loss of brain tissue, appears black due to autofluorescence of the tissue due to eye pigment, and was measured in the central brain and in the optic lobes using Fiji ImageJ Software (ImageJ v2.16.0/1.54p) [52]. For statistical analysis, we used the D’Agostino–Pearson test and the non-parametric Mann–Whitney test (GraphPad Prism v6.07; GraphPad Software Inc., San Diego, CA, USA).

## 5. Conclusions

In summary, our experiments show that SL extracts can provide resilience to age-related functional decline. The improvements in phototaxis from SLE, SLAq, and baicalin indicate that baicalin is mediating the beneficial functions. In contrast, only SLE and the wogon mix reduced sleep fragmentation, and this was only the case in males. This shows that the efficacy of SL is dependent on the extraction method and the specific behavior tested, and it can vary in males and females. Future experiments are needed to address whether this is also the case in mammals.

## Figures and Tables

**Table 1:** Concentration of quantified compounds in the two extracts.

Compound	µg/mg SLE	µg/mg SLAq
Baicalin	97.5358	91.9381
Wogonoside	1.1236	0.8678
Wogonin	0.0048	n.d.
Norwogonin	0.0074	n.d.
Apigenin	0.0013	n.d.
Scutellarein	0.0110	n.d.
Chrysin	0.0050	n.d.
Oroxylin A	0.0072	0.0012

**Table 2:** Tandem mass spectrometer (MS/MS) settings.

Compound	Retention Time (Minutes)	Quad 1 Mass	Quad 3 Mass	Depolarizing Potential (V)	Exit Potential (V)	Collision Energy (V)	Collision Cell Exit Potential (V)
Baicalin	2.82	447	271	100	15	30	10
Wogonin	4.8	285	270	100	10	30	5
Wogonoside	3.17	461	285	100	10	30	15
Chrysin	4.83	255	153	75	5	40	10
Oroxylin A	4.97	285	270	35	5	30	15
Norwogonin	3.65	271	169	75	15	40	10
Apigenin	3.5	271	153	50	5	40	20
Scutellarein	2.86	287	169	100	10	60	20

## Data Availability

The original contributions presented in this study are included in the article/Supplementary Materials. Further inquiries can be directed to the corresponding author.

## Supplementary Materials

The following supporting information can be downloaded at https://www.mdpi.com/article/10.3390/ijms27010461/s1.
