# Supplementary figures and images for: Scutellaria lateriflora Extract Supplementation Provides Resilience to Age-Related Phenotypes in Drosophila melanogaster
Source: Int J Mol Sci. 2026 Jan 1;27(1):461. doi: 10.3390/ijms27010461 (PMC12786168; doi:10.3390/ijms27010461)

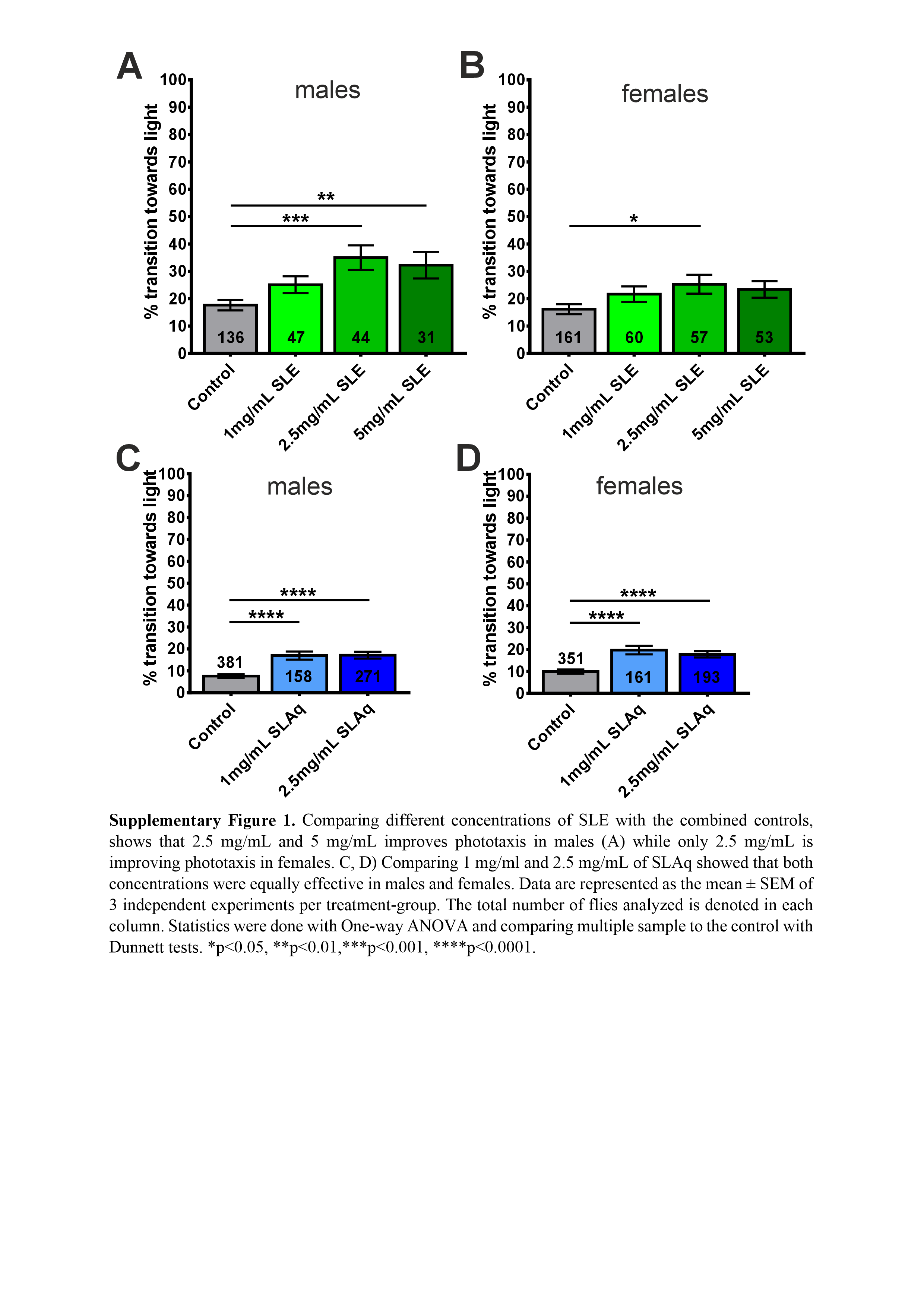

Supplement: Supplementary file 1 [file ijms-27-00461-s001.zip › ijms-4035315-supplementary.tif]
